# Adiponectin protects against myocardial ischemia–reperfusion injury: a systematic review and meta-analysis of preclinical animal studies

**DOI:** 10.1186/s12944-024-02028-w

**Published:** 2024-02-17

**Authors:** Hongyi Yue, Qunhui Zhang, Senhao Chang, Xinjie Zhao, Mengjie Wang, Wenhua Li

**Affiliations:** 1 Engineering Research Center of Tibetan Medicine Detection Technology, Ministry of Education, School of Medicine, Xizang Minzu University, Xianyang, 712082 Shaanxi China; 2 The First Affiliated Hospital, Department of Cardiology, Hengyang Medical School, University of South China, Hunan, 421001 China; 3 Hunan Provincial Key Laboratory of Multi-omics And Artificial Intelligence of Cardiovascular Diseases, University of South China, Hunan, 421001 China

**Keywords:** Adiponectin, Myocardial ischemia–reperfusion injury, Apoptosis, Oxidation, Inflammation, Animals

## Abstract

**Background:**

Myocardial ischemia–reperfusion injury (MIRI) is widespread in the treatment of ischemic heart disease, and its treatment options are currently limited. Adiponectin (APN) is an adipocytokine with cardioprotective properties; however, the mechanisms of APN in MIRI are unclear. Therefore, based on preclinical (animal model) evidence, the cardioprotective effects of APN and the underlying mechanisms were explored.

**Methods:**

The literature was searched for the protective effect of APN on MIRI in six databases until 16 November 2023, and data were extracted according to selection criteria. The outcomes were the size of the myocardial necrosis area and hemodynamics. Markers of oxidation, apoptosis, and inflammation were secondary outcome indicators. The quality evaluation was performed using the animal study evaluation scale recommended by the Systematic Review Center for Laboratory animal Experimentation statement. Stata/MP 14.0 software was used for the summary analysis.

**Results:**

In total, 20 papers with 426 animals were included in this study. The pooled analysis revealed that APN significantly reduced myocardial infarct size [weighted mean difference (WMD) = 16.67 (95% confidence interval (CI) = 13.18 to 20.16, *P* < 0.001)] and improved hemodynamics compared to the MIRI group [Left ventricular end-diastolic pressure: WMD = 5.96 (95% CI = 4.23 to 7.70, *P* < 0.001); + dP/dtmax: WMD = 1393.59 (95% CI = 972.57 to 1814.60, *P* < 0.001); -dP/dtmax: WMD = 850.06 (95% CI = 541.22 to 1158.90, *P* < 0.001); Left ventricular ejection fraction: WMD = 9.96 (95% CI = 7.29 to 12.63, *P* < 0.001)]. Apoptosis indicators [caspase-3: standardized mean difference (SMD) = 3.86 (95% CI = 2.97 to 4.76, *P* < 0.001); TUNEL-positive cells: WMD = 13.10 (95% CI = 8.15 to 18.05, *P* < 0.001)], inflammatory factor levels [TNF-α: SMD = 4.23 (95% CI = 2.48 to 5.98, *P* < 0.001)], oxidative stress indicators [Superoxide production: SMD = 4.53 (95% CI = 2.39 to 6.67, *P* < 0.001)], and lactate dehydrogenase levels [SMD = 2.82 (95% CI = 1.60 to 4.04, *P* < 0.001)] were significantly reduced. However, the superoxide dismutase content was significantly increased [SMD = 1.91 (95% CI = 1.17 to 2.65, *P* < 0.001)].

**Conclusion:**

APN protects against MIRI via anti-inflammatory, antiapoptotic, and antioxidant effects, and this effect is achieved by activating different signaling pathways.

**Supplementary Information:**

The online version contains supplementary material available at 10.1186/s12944-024-02028-w.

## Introduction

Ischemic heart disease (IDH) is a common cardiovascular disease that places substantial burdens on society [1]. Timely restoration of blood flow in the ischemic area is crucial to restore oxygen and nutrient supply, which is important for saving the damaged myocardium. The most effective therapeutic strategies are coronary artery bypass grafting and percutaneous coronary intervention [2]. However, this leads to disturbances in myocardial function and electrical activity, structural damage, and exacerbation of myocardial necrosis, a phenomenon termed myocardial ischemia–reperfusion injury (MIRI) [3, 4]. Treatment for MIRI is scarce, and the pathogenesis is complex. Although the exact mechanisms are not fully understood, research has indicated a potential link among oxidative stress, apoptosis, inflammation, and their mechanisms of action [5–7].

Adiponectin (APN) is an adipokine responsible for regulating the metabolism of glucose and lipids [8]. APN also has anti-inflammatory, antithrombotic, antioxidative stress, and antiatherosclerotic properties [9, 10]; it may also be involved in myocardial protection against MIRI. Braun et al. found that APN knockout mice have more severe myocardial infarctions after ischemia–reperfusion than wild-type mice [11]. Kim et al. demonstrated that the high incidence of ischemic heart disease in the diabetic population is strongly associated with a decrease in APN levels [12]. These studies suggest that APN has cardioprotective effects against MIRI.

To date, few clinical studies have revealed the protective effect of APN on MIRI, and most of the findings are limited to animal studies. Animal models have limitations in that they cannot reproduce the complex pathophysiological processes in humans, and conclusions are generally drawn from relatively small independent samples, which can be biased. Nevertheless, animal experiments are necessary, and a well-designed meta-analysis can provide compelling evidence while minimizing bias. Therefore, the present study hypothesized that APN may mitigate MIRI. A meta-analysis of relevant studies and summarizing possible mechanisms will accelerate the translation to clinical studies and provide a basis for MIRI treatment.

## Methods

The present study adhered to the requirements of the Cochrane Handbook Systematic Reviews of Interventions (version 6.2) and the Preferred Reporting Items for Systematic Reviews and Meta-Analyses (PRISMA) 2020 statement [13] (Supplementary File 1). This study was registered in the Prospective Registry for Systematic Reviews platform (PROSPERO), where any modifications can be found (ID: CRD42023433357).

### Literature search

A search of Web of Science, EmBase, SinoMed, PubMed, CNKI, and WanFang databases was used to collect the relevant literature on the beneficial effect of APN on MIRI without limitation of the year, language, type of article, or research object. The search time was from the creation of the database to 16 November 2023. The following search terms were utilized: [(adiponectin) OR (acrp30) OR (adipokines) OR (apM1 protein)] AND [(myocardial ischemia–reperfusion injury) OR (ischemic heart disease) OR (myocardial ischemia) OR (myocardial reperfusion)]. Citations from relevant literature were tracked to ensure the integrity of the included studies as much as possible.

### Eligibility criteria

The inclusion criteria were as follows: (1) the MIRI model was established by using appropriate methods; (2) the treatment group was injected with exogenous recombinant APN (proteolytic cleavage product of APN), and the MIRI group was given normal saline or a carrier without the drug (there were no requirements for the mode of administration, drug action time, or the dose of the two groups); (3) the object of study was animals; (4) the outcomes were the size of the myocardial necrosis area and hemodynamic monitoring indicators; and (5) markers of oxidation, apoptosis, and inflammation were used as secondary outcome indicators. The exclusion criteria were as follows: (1) duplicate publications; (2) editorials, conferences, abstracts, case reports, meta-analysis, and technical reports; (3) in vitro and clinical studies; (4) lack of intervention or control group modeling; (5) use of the same set of research data; (6) APN was not the only intervention; and (7) incomplete data.

### Data extraction

Two authors (HY and QZ) examined each study based on selection criteria and extracted relevant data. The following information was extracted: (1) author and year in which the article was published; (2) type, sex, weight, and weekly age of laboratory animals; (3) type of narcotic drug used; (4) methodology for the construction of the MIRI model; (5) APN treatment information, including dose, time, and method of administration; (6) time of ischemia and reperfusion; (7) presence of comorbidities; (8) outcome indicators and corresponding *p*-values; and (9) signaling pathways involved and corresponding mechanisms. When the sample size given by the study was not a definite value, the minimum sample size was taken to ensure accuracy. When there were differences in the dose and duration of action of APN in the intervention group, the differences were combined into a single treatment group according to the formula recommended by the Cochrane Handbook [14, 15]. For articles that only display data graphically, the data were obtained using GetData graph digitizer 2.22 (http://getdata-gra ph-digitizer.com/) based on the method of Chen et al. [16].

### Quality evaluation

Quality evaluation of the 20 studies was performed by HY and QZ using the risk assessment scale recommended by the Systematic Review Center for Laboratory animal Experimentation (SYRCLE) statement according to the methodology of Hooijmans et al. [17]. A third author (WH) checked the raw data for corrections. The scale evaluates the following components: (A) whether sequence generation was described in detail; (B) whether animals were characterized in detail at baseline; (C) sufficiency of allocation sequence hiding; (D) whether the animals were randomly captive; (E) whether the researcher was blinded; (F) whether the outcome measures were randomized; (G) whether the researcher was blinded at the time of outcome assessment; (H) whether incompleteness of outcome data was adequately addressed; (I) whether the results were selectively reported; and (J) whether other sources of bias were described. The included studies were visualised using RevMan 5.4.1. A third author (WH) adjudicated when there was disagreement. At the same time, the Grading of Recommendations, Assessment, Development, and Evaluations (GRADE) system was used to assess the quality of each outcome [18].

### Statistical analysis

Stata/MP 14.0 software was utilized to generate the forest plots and analyze all data. When *P* < 0.05, the analysis was considered meaningful. The mean ± standard deviation was used to represent the parameters of the forest map. For studies expressed as the median and interquartile range (e.g., Kondo, 2010), data were extracted after calculations based on the method stated by Wan et al. [19, 20]. Regarding the forest plots [21], the standardized mean difference (SMD) was used for pooled analysis when the methods or units of measurement were inconsistent, and the value of the confidence interval (CI) was set at 95%. When the measurement method or unit was consistent, the analysis was combined using the weighted mean difference (WMD). Because the number of animals in preclinical studies was generally small, a hedge was selected for statistical analysis of the effect size of SMD [22]. The I squared (*I*^*2*^) statistic assessed the variability among the studies involved. The DerSimonian and Laird method was used to develop a random-effects model, and the inverse variance method was used to develop a fixed-effects model. When *I*^*2*^ > 50% or *P* < 0.10, which indicated significant heterogeneity, the analyses were pooled using random-effect models. When *I*^*2*^ < 50% or *P* > 0.10, the choice of effect model was fixed [23]. To investigate potential variations in the studies, predefined subgroups were examined for primary outcomes displaying significant heterogeneity. The predefined subgroups mainly included various species, different methods of APN administration, and the presence or absence of comorbidities. Analyses used Begg's test, Egger's test [24], and funnel plots [25] to determine whether the included studies were biased. Sensitivity analyses determined whether the robustness of the included studies was sufficient. The approach was to exclude one study at a time and assess its impact on the overall outcome. When there were fewer than three articles on outcome measures, they were not analyzed.

## Results

### Study inclusion

**Fig. 1:**
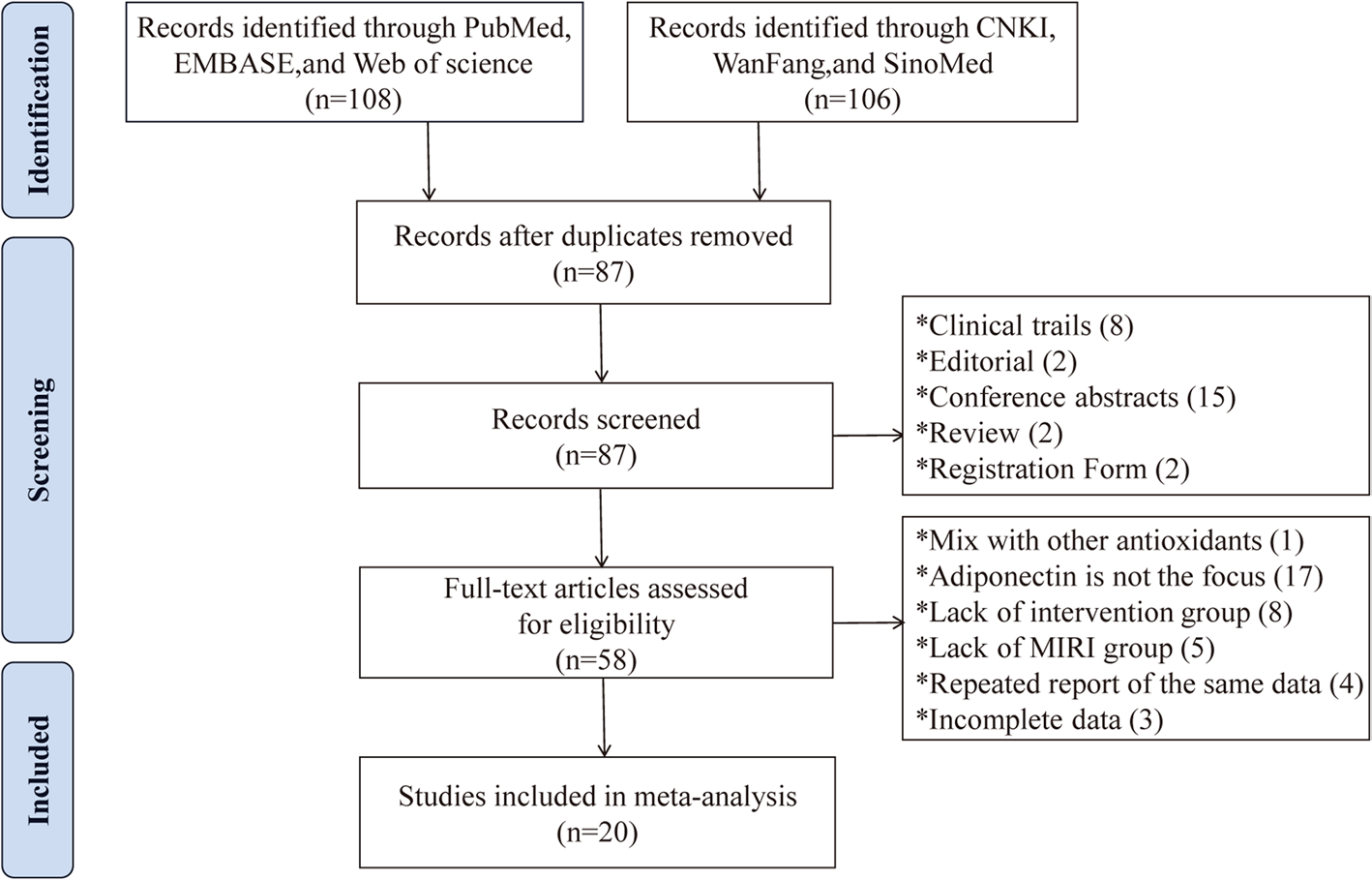
PRISMA Literature Search Flowchart

According to the search strategy, 214 articles were identified from six databases, including 106 articles in Chinese and 108 in English. First, 127 duplicate articles were removed using NoteExpress, and the abstracts and titles of the remaining 87 articles were read by HY and QZ, respectively. In total, 8 clinical studies, 2 editorials, 2 reviews, 15 conference abstracts, and 2 registration forms for scientific and technological achievements were excluded. The remaining 58 articles were read, and 38 articles were excluded due to the following reasons: 18 articles were excluded because APN was not the primary study subject or was mixed with other antioxidants; 13 articles were excluded due to the lack of intervention/MIRI group modeling; 4 articles were excluded due to the use of the same data set; and 3 articles were excluded due to incomplete data. Finally, 20 articles that met the requirements were included [26–45]. The process of searching for literature is depicted in Fig. 1.

### Features of the included studies

In total, 20 papers and 27 study cohorts (seven papers involving two study cohorts [26, 28, 32, 33, 37, 39, 41]), including 10 in English and 10 in Chinese, were included, with 426 animals (9 studies used rats [30, 31, 34, 36, 38, 42–45], 10 studies used mice [26–28, 32, 33, 35, 37, 39–41], and 1 study used pigs [29]). Regarding the MIRI model, one model was generated by the Langendorff cardiac perfusion system, and the remaining models were generated by coronary artery ligation. The duration of myocardial ischemia was usually 0.5/3 h; however, in five papers, the duration was 0.75 h. The reperfusion time was 24 h in nine studies, 48 h in one study, and 1–3 h in the remaining studies, of which 3 h accounted for the most significant proportion. APN pretreatment was primarily intravenous (IV) or intraperitoneal (IP), while pretreatment ranged from 2 min before surgery to 3 days before surgery. Regarding the dose of administration, the doses differed among studies. The anesthetics were primarily isoflurane and sodium pentobarbital; however, one study used urethane, and another used chloral hydrate. Fourteen studies had no disease other than MIRI [26–30, 34–37, 40–43, 45], while six studies considered comorbidities [31–33, 38, 39, 44]. Other basic features are shown in Table 1.

**Table 1:** Characteristic table of the literature

Author and Year	Species and gender	Week old and Weight	Anesthetic	Model and method	Treatment group		MI/RI Time	Comorbidity	Outcomes and P-Value	Mechanisms
					**APN and Duration**	**Administration**				
Shibata et al. 2005 [26]	Queue a (WT): C57BL/6 mices, ND	10 to 12 weeks, ND	Pent	MIRI, ligation of LAD	2 × 10^8^ PFU, 3 d	IV	0.5 h/48 h	no	1. Infarct size: *P* < 0.05 2. Apoptotic rate: *P* < 0.05 3. LVEDP: *P* < 0.05 4. + dP/dtmax: *P* < 0.05 5. -dP/dtmax: *P* < 0.05 6. TNF-α: *P* < 0.05	AMPK↑/COX-2↑/apoptosis↓/proinflammatory cytokine↓
	Queue b (APN-KO): C57BL/6 mices, ND	10 to 12 weeks, ND	Pent	MIRI, ligation of LAD	2 × 10^8^ PFU, 3 d	IV	0.5 h/48 h	no	1. Infarct size: *P* < 0.01 2. Apoptotic rate: *P* < 0.05 3. TNF-α: *P* < 0.01	
Tao et al. 2007 [27]	Adult mices, male	ND	Isoflurane	MIRI, ligation of LAD	1 to 4 μg/g, 10 min	IP	0.5 h/3 h, 24 h	no	1. Infarct size: *P* < 0.01 2. Apoptotic rate: *P* < 0.01 3. LVEDP: *P* < 0.01 4. + dP/dtmax: *P* < 0.01 5. -dP/dtmax: *P* < 0.01 6. Caspase-3: *P* < 0.01 7. Superoxide production: *P* < 0.01	iNOS↓/gp91^phox protein↓/oxidative or nitrative stress↓
Wang et al. 2009 [28]	Queue a (WT): D157A mices, male	ND	Isoflurane	MIRI, ligation of LAD	2 μg/g, 10 min	IP	0.5 h/3 h, 24 h	no	1. Infarct size: *P* < 0.01 2. Apoptotic rate: *P* < 0.01 3. LVEDP: *P* < 0.05 4. + dP/dtmax: *P* < 0.05 5. -dP/dtmax: *P* < 0.05 6. Caspase-3: *P* < 0.01 7. Superoxide production: *P* < 0.01 8. LDH: *P* < 0.01	apoptosis↓/oxidative or nitrative stress↓ (Note: the antioxidative and antinitrative effects of APN in MIRI hearts are not mediated by AMPK)
	Queue b (AMPK-DN): D157A mices, male	ND	Isoflurane	MIRI, ligation of LAD	2 μg/g, 10 min	IP	0.5 h/3 h, 24 h	no	1. Infarct size: *P* < 0.01 2. Apoptotic rate: *P* < 0.01 3. LVEDP: *P* < 0.05 4. + dP/dtmax: *P* < 0.05 5. -dP/dtmax: *P* < 0.05 6. Caspase-3: *P* < 0.05 7. Superoxide production: *P* < 0.05 8. LDH: *P* < 0.01	
Kondo et al. 2010 [29]	Yorkshire-Duroc pigs, female	2 to 3 months, 29.55 to 31.95 kg	Pent	MIRI, ligation of LAD	0.03 μg/kg, 10 min	Arterial administration	0.75 h/24 h	no	1. Infarct size: *P* < 0.05 2. Apoptotic rate: *P* < 0.01 3. LVEDP: *P* < 0.05 4. + dP/dtmax: *P* < 0.05 5. -dP/dtmax: *P* < 0.05 6. TNF-α: *P* < 0.05	inflammation↓/apoptosis↓/oxidative stress↓
Wang et al. 2010 [30]	SD rats, male	8 to 10 weeks, 180 to 220 g	Urethane	MIRI, ligation of LAD	60 ng/g, 2 min	IV	0.5 h/1 h	no	1. Caspase-3: *P* < 0.01	AMPK↑/PPAR-γ↑/apoptosis↓
Chen 2011 [31]	SD rats, male	ND, 220 to 250 g	Pent	MIRI, ligation of LAD	60/120/180 ng/g, 30 min	IV	0.75 h/3 h	DM	1. Infarct size: *P* < 0.01 2. + dP/dtmax: *P* < 0.01 3. -dP/dtmax: *P* < 0.01 4. Caspase-3: *P* < 0.01 5. SOD: *P* < 0.01	NOS↑/SOD↑/oxidative stress↓
Liu et al. 2011 [32]	Queue a (WT): C57B1/6 J mices, male	8 to 12 weeks, ND	Isoflurane	MIRI, ligation of LAD	0.5 mg/kg, 10 min	IV	0.5 h/3 h	Trauma	1. Infarct size: *P* < 0.01 2. + dP/dtmax: *P* < 0.01 3. Caspase-3: *P* < 0.01 4. Superoxide production: *P* < 0.05	apoptosis↓/oxidative or nitrative stress↓
	Queue b(APN-KO): C57B1/6 J mices, male	8 to 12 weeks, ND	Isoflurane	MIRI, ligation of LAD	0.5 mg/kg, 10 min	IV	0.5 h/3 h	Trauma	1. Infarct size: *P* < 0.01 2. + dP/dtmax: *P* < 0.01	
Ma et al. 2011 [33]	Queue a (DM1d): Swiss mices, ND	6 to 8 weeks, ND	Isoflurane	MIRI, ligation of LAD	2 μg/g, 10 min	IP	0.5 h/3 h, 24 h	DM	1. Infarct size: *P* > 0.05 2. Apoptotic rate: *P* > 0.05 3. Caspase-3: *P* > 0.05 4. LDH: *P* > 0.05	AdipoR1↑/AMPK↑/LDH↓/apoptosis↓
	Queue b (DM7d): Swiss mices, ND	6 to 8 weeks, ND	Isoflurane	MIRI, ligation of LAD	2 μg/g, 10 min	IP	0.5 h/3 h, 24 h	DM	1. Infarct size: *P* < 0.01 2. Apoptotic rate: *P* < 0.01 3. Caspase-3: *P* < 0.01 4. LDH: *P* < 0.01	
Chen et al. 2011 [34]	SD rats, male	ND, 220 to 250 g	Pent	MIRI, ligation of LAD	180 ng/g, 30 min	IV	0.75 h/3 h	no	1. LVEDP: *P* < 0.05 2. LDH: *P* < 0.01	apoptosis↓/oxidative stress↓
Gao et al. 2012 [35]	C57 mices, male	4 to 6 weeks, 20 to 25 g	Isoflurane	MIRI, ligation of LAD	0.5 mg /kg, 10 min	IP	0.5 h/3 h, 24 h	no	1. LVEF: *P* < 0.05 2. Caspase-3: *P* < 0.05	ND
Guo 2013 [36]	SD rats, male	7 to 8 weeks, 220 to 250 g	Pent	MIRI, ligation of LAD	1 μg/kg, 10 min	IV	0.75 h/3 h	no	1. Infarct size: *P* < 0.05 2. Apoptotic rate: *P* < 0.05 3. LVEDP: *P* < 0.05 4. + dP/dtmax: *P* < 0.05 5. -dP/dtmax: *P* < 0.05 6. Caspase-3: *P* < 0.05 7. LDH: *P* < 0.05	apoptosis↓/LDH↓/CK-MB↓/arrhythmia↓
Zhang et al. 2013 [37]	Queue a (Scramble): D157A mices, male	ND	Isoflurane	MIRI, ligation of LAD	2 μg/g, 20 min	IP	0.5 h/3 h, 24 h	no	1. Infarct size: *P* < 0.01 2. LVEDP: *P* < 0.01 3. + dP/dtmax: *P* < 0.01 4. LVEF: *P* < 0.05 5. Caspase-3: *P* < 0.01 6. Apoptotic rate: *P* < 0.01 7. Superoxide production: *P* < 0.01	PKA activity↑/oxidative stress↓/IKK, IκB↓/NF-κB↓
	Queue b (PKA-KO): D157A mices, male	ND	Isoflurane	MIRI, ligation of LAD	2 μg/g, 20 min	IP	0.5 h/3 h, 24 h	no	1. Infarct size: *P* < 0.05 2. LVEDP: *P* < 0.05 3. + dP/dtmax: *P* < 0.05 4. LVEF: *P* > 0.05 5. Caspase-3: *P* > 0.05 6. Apoptotic rate: *P* > 0.05 7. Superoxide production: *P* < 0.01	
Guo et al. 2014 [38]	SD rats, ND	ND, 220 to 250 g	Pent	MIRI, ligation of LAD	1 μg/kg, 10 min	IV	0.75 h/3 h	DM	1. Infarct size: *P* < 0.05 2. Caspase-3: *P* < 0.05 3. SOD: *P* < 0.05	apoptosis↓/oxidative stress↓
Song et al. 2014 [39]	Queue a (WT): C57B1/6 J mices, ND	8 to 10 weeks, ND	Isoflurane	MIRI, ligation of LAD	2 μg/g, 7 d	IP	0.5 h/3 h, 24 h	renal failure	1. Infarct size: *P* < 0.05 2. Apoptotic rate: *P* < 0.01 3. Caspase-3: *P* < 0.05 4. LVEF: *P* < 0.01	AdipoR1↑/AMPK↑/iNOS↓/apoptosis↓/oxidative or nitrative stress↓
	Queue b (APN-KO): C57B1/6 J mices, ND	8 to 10 weeks, ND	Isoflurane	MIRI, ligation of LAD	2 μg/g, 7 d	IP	0.5 h/3 h, 24 h	renal failure	1. Infarct size: *P* < 0.05 2. Apoptotic rate: *P* < 0.01 3. Caspase-3: *P* < 0.05 4. LVEF: *P* < 0.01	
Wang et al. 2014 [40]	C57BL/6 J mices, male	8 to 10 weeks, ND	Isoflurane	MIRI, ligation of LAD	2 μg /g, 10 min	IP	0.5 h/3 h, 24 h	no	1. Infarct size: *P* < 0.01 2. LVEF: *P* < 0.01	increased APPL1 expression
Gao et al. 2015 [41]	Queue a (WT): C57BL/6 mices, male	6 to 8 weeks, 20 to 25 g	Isoflurane	MIRI, ligation of LAD	0.5 mg/kg, 10 min	IP	0.5 h/3 h, 24 h	no	1. Infarct size: *P* < 0.01 2. Apoptotic rate: *P* < 0.05 3. LVEDP: *P* < 0.05 4. + dP/dtmax: *P* < 0.05 5. -dP/dtmax: *P* < 0.05 6. Caspase-3: *P* < 0.05 7. LVEF: *P* < 0.05	AMP-activated protein kinase phosphorylation↑/apoptosis↓/TNF-α↓
	Queue b (APN-KO): C57BL/6 mices, male	6 to 8 weeks, 20 to 25 g	Isoflurane	MIRI, ligation of LAD	0.5 mg/kg, 10 min	IP	0.5 h/3 h,24 h	no	1. Infarct size: *P* < 0.05 2. Apoptotic rate: *P* < 0.05 3. Caspase-3: *P* < 0.01 4. LVEF: *P* < 0.05	
Zhao et al. 2016 [42]	SD rats, ND	ND, 220 to 280 g	Chloral hydrate	MIRI, ligation of LAD	1.8 μg/g, 20 min	IV	0.5 h/2 h	no	1. LDH: *P* < 0.05 2. MDA: *P* < 0.05 3. SOD: *P* < 0.05	Lipid peroxidation↓/free radical scavenging↑
Potenza et al. 2019 [43]	SD rats, male	ND, 250 to 300 g	Pent	MIRI, Langendorff perfusion system	3 μg/mL, 3.5 h	Arterial administration	0.5 h/3 h	no	1. Infarct size: *P* < 0.05 2. LVEDP: *P* < 0.01	AMPK, LKB1↑/ eNOS↑/SIRT-1↑
Xing et al. 2020 [44]	SD rats, male	8 to 10 weeks, 250 to 300 g	Pent	MIRI ligation of LAD	5 μg /g, 15 min	IP	0.5 h/2 h	DM	1. Infarct size: *P* < 0.05	SphK1↓/S1P↓/phosphorylation of NF-κB↓
Wang et al. 2022 [45]	SD rats, male	4 weeks, 180 to 220 g	Pent	MIRI ligation of LAD	5 μg/g, 15 min	IP	0.5 h/2 h	no	1. LDH: *P* < 0.05	MIF↑/autophagy↓

*Pent* Pentobarbital sodium, *PFU* plaque-forming units, *IV* Intravenous injection, *IP* Intraperitoneal injection, *IS* infarct size, *AAR* area at risk, *ND* no description, *WT* wild-type, *APN-KO* APN gene knockout, *AMPK-DN* a mutant AMPKα2 subunit, *MIRI* myocardial ischemia/reperfusion injury, *d* day, *h* hour, *min* minute, *LAD* left anterior descending branch, *SD* Sprague–Dawley, *COX-2* Cyclooxygenase-2, *iNOS* inducible NO synthase, *gp91^phox* nicotinamide adenine dinucleotide phosphate-oxidase, *LVEDP* left ventricular end-diastolic pressure, + *dP/dtmax* maximum rate of left ventricular pressure rise, *-dP/dtmax* maximum rate of left ventricular pressure decrease, *LVEF* left ventricular ejection fraction, *DM* diabetes mellitus, *SOD* superoxide dismutase, *NO* nitric oxide, *NOS* nitric oxide synthase, *PKA-KO* Protein kinase A knockout, *CK-MB* creatine kinase isoenzymes, *APPL1* Recombinant Adiponectin Receptor 1, *eNOS* endothelial nitric oxide synthase, *LKB1* Liver kinase B1, *SIRT-1* sirtuin-1, *SphK1* Sheath Amino Acid Kinase 1, *S1P* Sphingosine-1-phosphate, *TNF-α* tumor necrosis factor alpha, *IHC* immunohistochemical staining, *PPAR-γ* Peroxisome proliferator activated receptor, *MIF* migration inhibitory factor

### Quality assessment of research

**Fig. 2:**
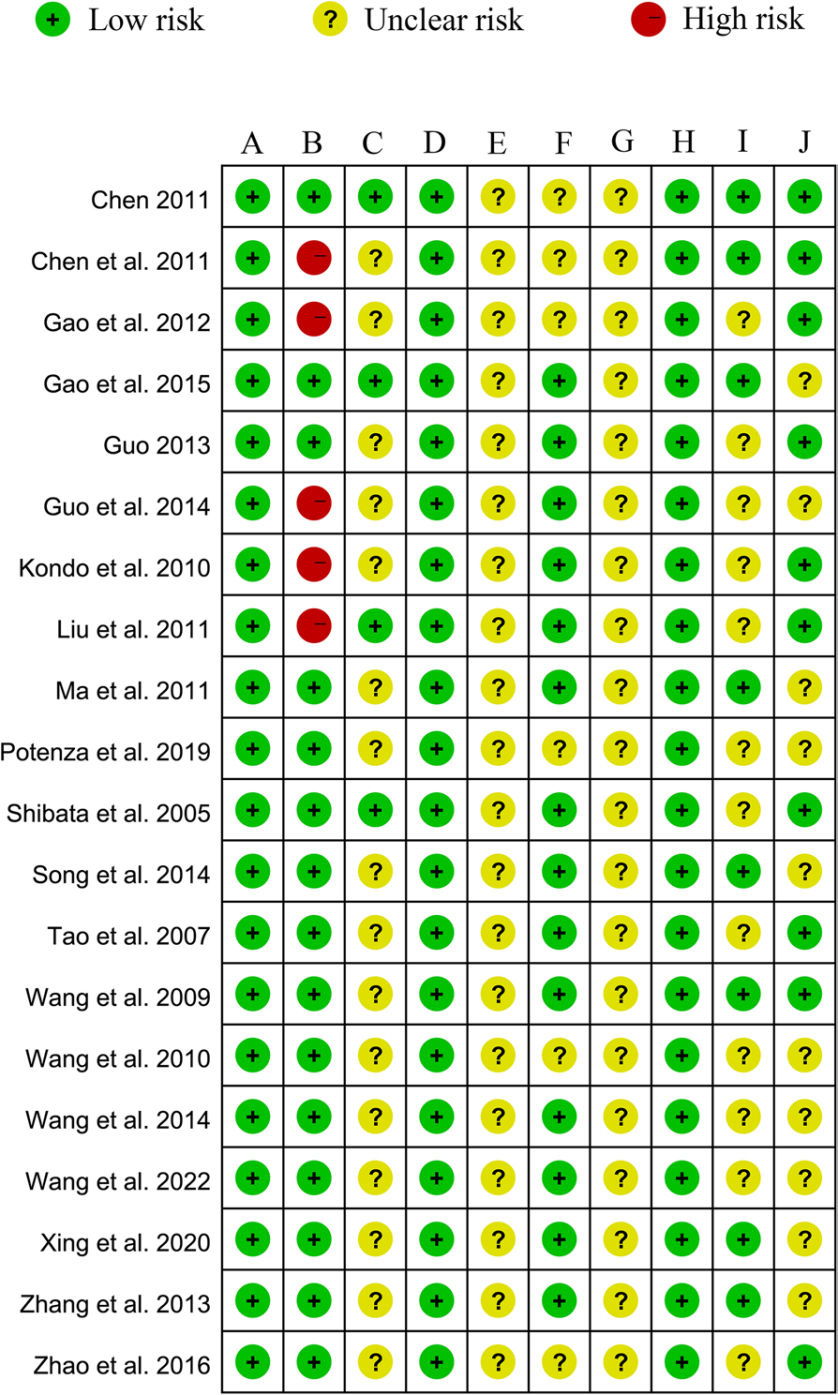
Quality evaluation of 20 animal literatures (SYRCLE tool). **A** Formation of sequences; **B** Initial characteristics of the animal; **C** Sequence hiding; **D** Randomness of animal feeding; **E** Personnel blinding; **F**: Randomness of outcome measurement; **G** Outcome assessment bias; **H** Integrity of result data; **I** Biased results; **J** Other biases

The content of 20 articles was independently assessed according to the 10-item systematic review scale for animal experiments recommended by the SYRCLE statement. Sequence generation was described in detail in all studies. Animals were randomly assigned to rearing, and the incompleteness of the resulting data was adequately addressed in all studies. None of the studies provided a detailed description of the blinding of researchers or blinding during outcome assessment. Five studies did not describe (rated as high risk) the baseline characteristics of the animals in detail [29, 32, 34, 35, 38], and six studies did not adequately describe whether or not they were randomized at the time of the outcome measure [30, 31, 34, 35, 42, 43]. Moreover, nine studies adequately illustrated the limitations of the study [26–29, 32, 34–36, 42]. Overall, the risk of bias was unclear in most studies. Only a few studies were at high risk, and there was a relatively high level of confidence in the evidence for inclusion. Other specific details are provided in Fig. 2.

### Evidence quality evaluation

Eleven outcome indicators were evaluated using the GRADE approach. Of these, 27.27% (3/11) were deemed to be of moderate quality, 36.36% (4/11) were considered to be of low quality, and the rest were rated as critically low quality (Table 2). It is important to note that inconsistencies and risk of bias were the main reasons for the downgrading of the evidence. In addition, due to significant heterogeneity in some results, we downgraded the quality of these results.

**Table 2:** A GRADE summary of the protective effect of APN on MIRI

Outcomes	Summary of finding		Relative effect (95% CI)	Limitations	Inconsistency	Indirectness	Imprecision	Publication bias	Quality
	MIRI group	APN group							
Infarct size	171 animals in 15 studies	169 animals in 15 studies	WMD, 16.67 (13.18, 20.16)	+	−	+	+	+	⨁⨁⨁◯, Moderate
LVEDP	99 animals in 9 studies	98 animals in 9 studies	WMD, 5.96 (4.23, 7.70)	−	−	+	+	−	⨁◯◯◯, Critically low
+ dp/dtmax	107 animals in 9 studies	107 animals in 9 studies	WMD, 1393.59 (972.57, 1814.60)	+	−	+	+	−	⨁⨁◯◯, Low
-dp/dtmax	68 animals in 7 studies	68 animals in 7 studies	WMD, 850.06 (541.22, 1158.90)	+	−	+	+	+	⨁⨁⨁◯, Moderate
LVEF	68 animals in 5 studies	68 animals in 5 studies	WMD, 9.96 (7.29, 12.63)	+	−	+	+	+	⨁⨁⨁◯, Moderate
Caspase-3	126 animals in 12 studies	126 animals in 12 studies	SMD, 3.86 (2.97, 4.76)	−	−	+	+	−	⨁◯◯◯, Critically low
Apoptotic rate	95 animals in 9 studies	95 animals in 9 studies	WMD, 13.10 (8.15, 18.05)	−	−	+	+	+	⨁⨁◯◯, Low
SOD	24 animals in 3 studies	24 animals in 3 studies	SMD, 1.91 (1.17, 2.65)	−	+	+	+	−	⨁⨁◯◯, Low
LDH	70 animals in 6 studies	70 animals in 6 studies	SMD, 2.82 (1.60, 4.04)	−	−	+	+	−	⨁◯◯◯, Critically low
TNF-α	13 animals in 2 studies	13 animals in 2 studies	SMD, 4.23 (2.48, 5.98)	−	+	+	+	−	⨁⨁◯◯, Low
Superoxide production	43 animals in 4 studies	43 animals in 4 studies	SMD, 4.53 (2.39, 6.67)	−	−	+	+	−	⨁◯◯◯, Critically low

* − *downgrade, + not downgrade, *LVEDP* Left ventricular end-diastolic pressure, + *dp/dtmax* Maximum rate of left ventricular pressure rise, *-dp/dtmax* Maximum rate of left ventricular pressure decrease, *LVEF* Left ventricular ejection fraction, *SOD* Superoxide dismutase, *LDH* lactate dehydrogenase, *SMD* standardized mean difference, *WMD* weighted mean difference

### Meta-analysis results

#### Myocardial infarction size

**Fig. 3:**
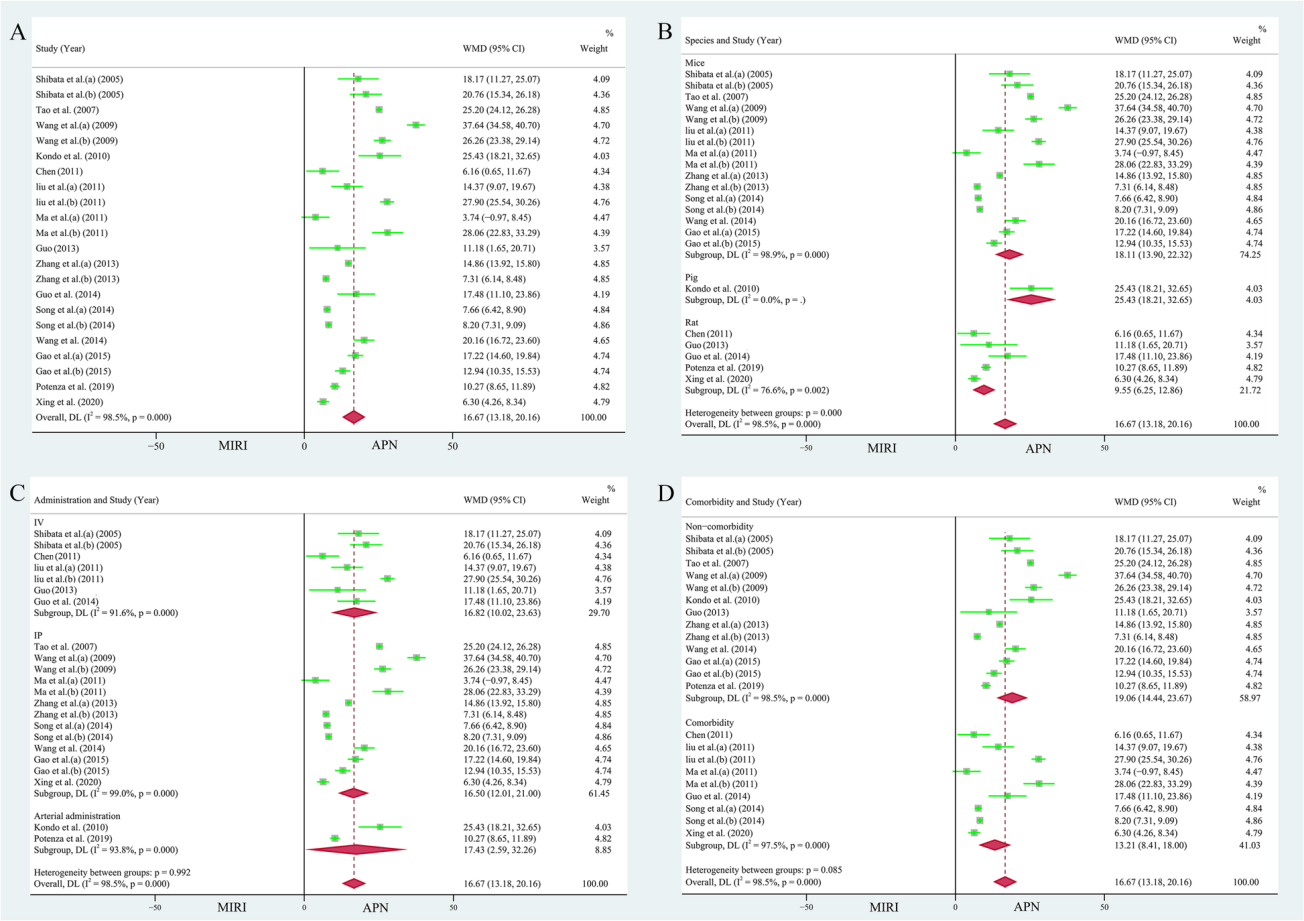
**A** Forest plot of the effect of APN on myocardial infarct size. Subgroup analyses of the effect of APN on myocardial infarct size included **B** different species, **C** different modes of administration, and **D** presence of comorbidities

Fifteen publications (22 study cohorts), including 340 animals, reported the relationship between APN and myocardial infarct size [26–29, 31–33, 36–41, 43, 44]. Due to the heterogeneity test results (*I*^*2*^ = 98.50%, *P* = 0.000), the random effects model was used for analysis. The area of myocardial infarction decreased by 16.67% due to the application of APN (WMD = 16.67, 95% CI = 13.18 to 20.16, *P* < 0.001, Fig. 3A). However, the funnel plot was not symmetrical (Supplementary Fig. 1A). Egger's test (*t* = 1.16, *P* = 0.261 > 0.05) and Begg's test (*Z* = 1.02, *P* = 0.310) showed no bias. Sensitivity analyses showed (Supplementary Fig. 1B) that no study significantly changed the total effect sizes and *I*^*2*^ values of the pooled analyses, indicating relatively stable results.

The studies were stratified according to species (mice: 246 animals in 16 studies, heterogeneity: *I*^*2*^ = 98.90%, *P* = 0.000, WMD = 18.11, 95% CI = 13.90 to 22.32, *P* < 0.001; rats: 83 animals in 5 studies, heterogeneity: *I*^*2*^ = 76.60%, *P* = 0.002, WMD = 9.55, 95% CI = 6.25 to 12.86, *P* < 0.001, Fig. 3B), different modes of administration (IV: 7 studies with 86 animals, heterogeneity: *I*^*2*^ = 91.60%, *P* = 0.000, WMD = 16.82, 95% CI = 10.02 to 23.63, *P* < 0.001; IP: 13 studies 230 animals, heterogeneity: *I*^*2*^ = 99.00%, *P* = 0.000, WMD = 16.50, 95% CI = 12.01 to 21.00, *P* < 0.001; arterial administration: 24 animals in 2 studies, heterogeneity: *I*^*2*^ = 93.80%, *P* = 0.000, WMD = 17.43, 95% CI = 2.59 to 32.26, *P* < 0.05, Fig. 3C), and presence of comorbidities (non-comorbidity: 204 animals in 13 studies, heterogeneity: *I*^*2*^ = 98.50%, *P* = 0.000, WMD = 19.06, 95% CI = 14.44 to 23.67, *P* < 0.001; comorbidity: 136 animals in 9 studies, heterogeneity: *I*^*2*^ = 97.50%, *P* = 0.000, WMD = 13.21, 95% CI = 8.41 to 18.00, *P* < 0.001, Fig. 3D), to further explore sources of heterogeneity. APN still significantly reduced the size of the infarcted myocardium in different subgroups, and the heterogeneity remained significant.

### Hemodynamics-related indicators

#### Left ventricular end-diastolic pressure (LVEDP)

**Fig. 4:**
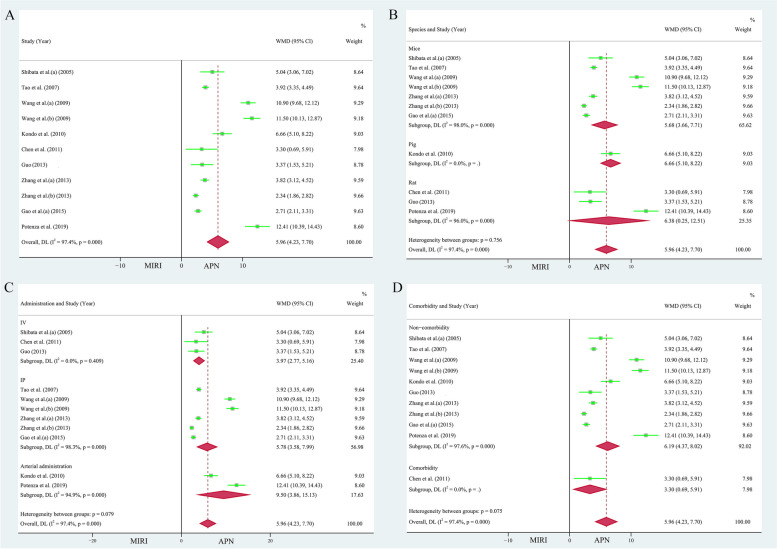
**A** Forest plot of the protective effect of APN on LVEDP. Subgroup analyses of the protective effect of APN on LVEDP included **B** different species, **C** different modes of administration, and **D** presence of comorbidities

Nine papers (11 study cohorts), containing 197 animals, reported the protective effect of APN on LVEDP [26–29, 34, 36, 37, 41, 43]. Due to the heterogeneity test result (*I*^*2*^ = 97.40%, *P* = 0.000), the individual effects model selection was randomized. The comprehensive analysis showed that APN had an excellent reduction of LVEDP by 5.96 mmHg compared to the MIRI cohort (WMD = 5.96, 95% CI = 4.23 to 7.70, *P* < 0.001, Fig. 4A). Egger's test (*t* = 2.52, *P* = 0.033 < 0.05) and the funnel plot (Supplementary Fig. 2A) showed publication skewing. Begg's test (*P* = 0.350, *Z* = 0.93) indicated bias. Sensitivity analysis indicated the results were stable and credible (Supplementary Fig. 2C).

The beneficial effect of APN on LVEDP was observed in the subgroups according to different species (mice: 138 animals in 7 studies, heterogeneity: *I*^*2*^ = 98.00%, *P* = 0.000, WMD = 5.68, 95% CI = 3.66 to 7.71, *P* < 0.001; rats: 45 animals in 3 studies, heterogeneity: *I*^*2*^ = 96.00%, *P* = 0.000, WMD = 6.38, 95% CI = 0.25 to 12.51, *P* < 0.05, Fig. 4B), different modes of administration (IV: 3 studies 42 animals, WMD = 3.97, 95% CI = 2.77 to 5.16, *P* < 0.001; IP: 6 studies with 128 animals, heterogeneity: *I*^*2*^ = 98.30%, *P* = 0.000, WMD = 5.78, 95% CI = 3.58 to 7.99, *P* < 0.001; arterial administration: 27 animals in 2 studies, heterogeneity: *I*^*2*^ = 94.90%, *P* = 0.000, WMD = 9.50, 95% CI = 3.86 to 15.13, *P* < 0.05, Fig. 4C), and the presence of comorbidities (non-comorbidity: 181 animals in 10 studies, heterogeneity: *I*^*2*^ = 97.60%, *P* = 0.000, WMD = 6.19, 95% CI = 4.37 to 8.02, *P* < 0.001, Fig. 4D). However, when stratifying the studies according to different modes of administration, the heterogeneity of the protective effect of APN on LVEDP was not significant in subgroup IV (*I*^*2*^ = 00.00%, *P* = 0.409).

### Maximum rate of left ventricular pressure rise (+ dp/dtmax)

**Fig. 5:**
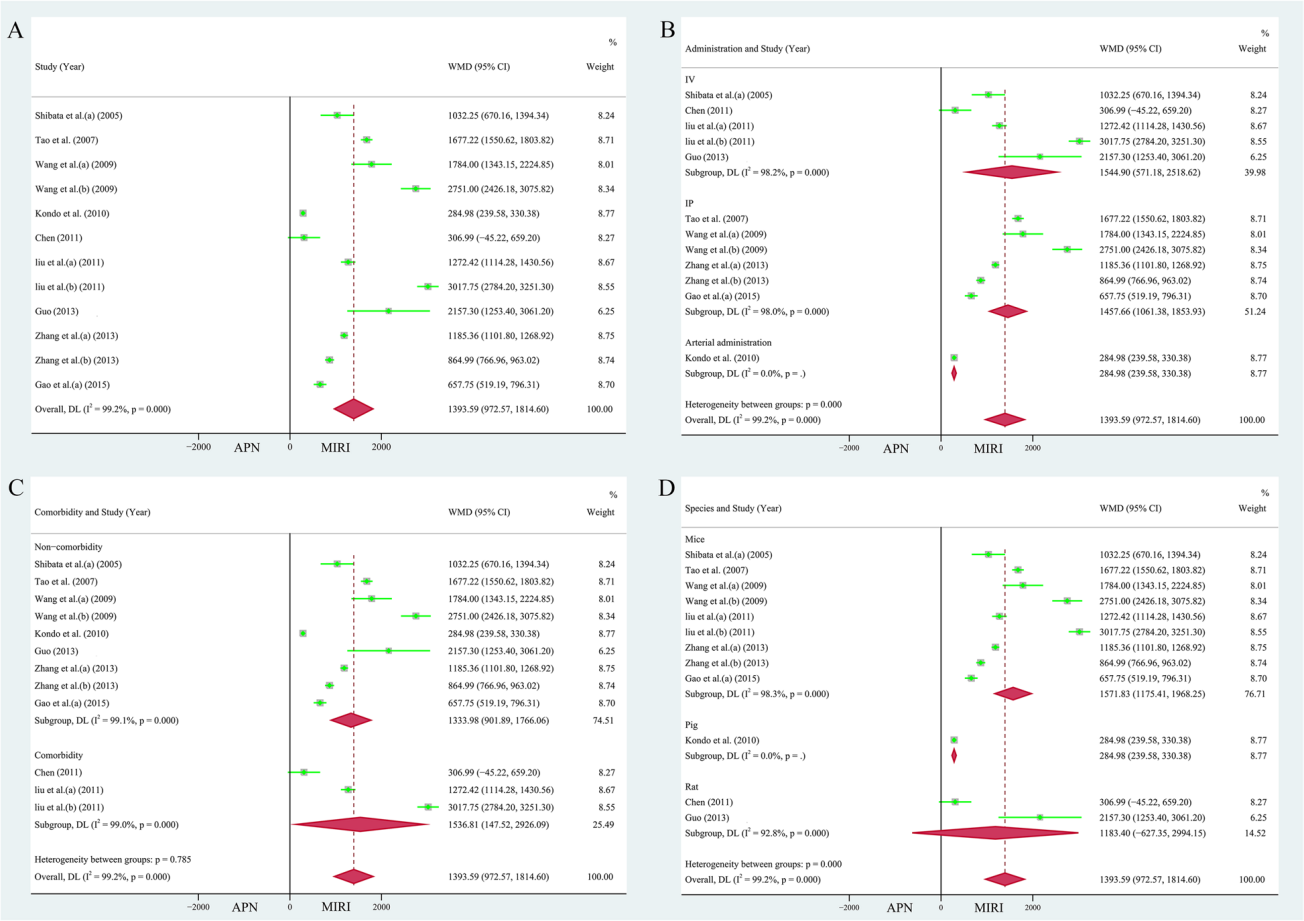
**A** Forest plot of the protective effect of APN on + dp/dtmax. Subgroup analyses of the protective effect of APN on + dp/dtmax included **B** different modes of administration, **C** presence of comorbidities, and **D** different species

Nine publications (12 study cohorts), containing 214 animals, reported the protective effect of APN on + dp/dtmax [26–29, 31, 32, 36, 37, 41]. The heterogeneity test result (*I*^*2*^ = 99.20%, *P* = 0.000) indicated that a random effects model was required for analysis. Pooled analysis showed a significant increase in + dp/dtmax due to APN administration compared to the MIRI cohort (WMD = 1393.59, 95% CI = 972.57 to 1814.60, *P* < 0.001, Fig. 5A). Egger's test (*t* = 2.71, *P* = 0.022 < 0.05) and the funnel plot (Supplementary Fig. 2B) indicated bias in the included studies. In addition, Begg's test (*P* = 0.945, *Z* = 0.07) indicated bias. Sensitivity analysis indicated the results were stable and credible (Supplementary Fig. 2D).

The beneficial effect of APN on + dp/dtmax was maintained when the studies were stratified according to different modes of administration (IV: 72 animals in 5 studies, heterogeneity: *I*^*2*^ = 98.20%, *P* = 0.000, WMD = 1544.90, 95% CI = 571.18 to 2518.62, *P* < 0.05; IP: 128 animals in 6 studies, heterogeneity: *I*^*2*^ = 98.00%, *P* = 0.000, WMD = 1457.66, 95% CI = 1061.38 to 1853.93, *P* < 0.001, Fig. 5B), and the presence of comorbidities (non-comorbidity: 168 animals in 9 studies, heterogeneity: *I*^*2*^ = 99.10%, *P* = 0.000, WMD = 1333.98, 95% CI = 901.89 to 1766.06, *P* < 0.001; comorbidity: 46 animals in 3 studies, heterogeneity: *I*^*2*^ = 99.00%, *P* = 0.000, WMD = 1536.81, 95% CI = 147.52 to 2926.09, *P* < 0.05, Fig. 5C). However, when the studies were stratified according to species, the protective effect of APN on + dp/dtmax was only statistically significant in mice (168 animals in 9 studies, heterogeneity: *I*^*2*^ = 98.30%, *P* = 0.000, WMD = 1571.83, 95% CI = 1175.41 to 1968.25, *P* < 0.001, Fig. 5D).

### Maximum rate of left ventricular pressure decrease (-dp/dtmax)

**Fig. 6:**
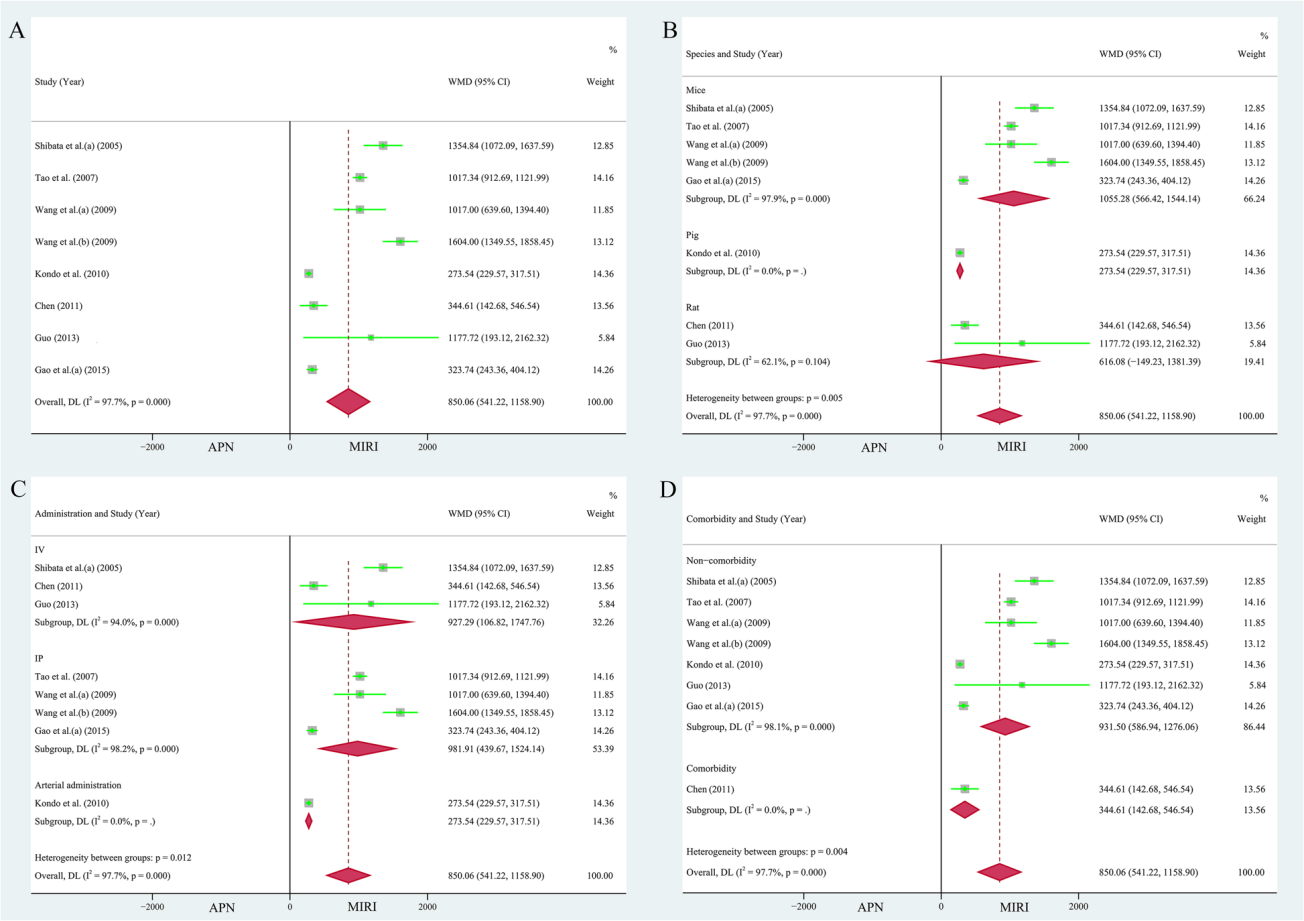
**A** Forest plot of the protective effect of APN on -dp/dtmax. Subgroup analyses of the protective effect of APN on -dp/dtmax included **B** different species, **C** different modes of administration, and **D** presence of comorbidities

Pooled analysis of seven publications (eight study cohorts) showed that the -dp/dtmax was significantly lower in the MIRI group compared to the APN administration group [26–29, 31, 36, 41], with a difference of 850.06 mmHg/s (n = 136, heterogeneity: *I*^*2*^ = 97.70%, *P* = 0.000, WMD = 850.06, 95% CI = 541.22 to 1158.90, *P* < 0.001, Fig. 6A). Although the funnel plot (Supplementary Fig. 3A) was asymmetrical, Egger's (*t* = 2.23, *P* = 0.067 > 0.05) and Begg's (*P* = 0.536, *Z* = 0.62) tests did not indicate bias. Sensitivity analysis indicated the results were robust and reliable (Supplementary Fig. 3C).

When stratifying the studies according to different species, the protective effect of APN on -dp/dtmax was only statistically significant in mice (46 animals in 5 studies, heterogeneity: *I*^*2*^ = 97.90%, *P* = 0.000, WMD = 1055.28, 95% CI = 566.42 to 1544.14, *P* < 0.001, Fig. 6B). In contrast, the protective effect of APN on -dp/dtmax, as well as the heterogeneity, remained significant when the studies were stratified according to the different modes of administration (IV: 42 animals in 3 studies, heterogeneity: *I*^*2*^ = 94.00%, *P* = 0.000, WMD = 927.29, 95% CI = 106.82 to 1747.76, *P* < 0.05; IP: 80 animals in 4 studies, heterogeneity: *I*^*2*^ = 98.20%, *P* = 0.000, WMD = 981.91, 95% CI = 439.67 to 1524.14, *P* < 0.001, Fig. 6C), and the presence of comorbidities (non-comorbidity: 120 animals in 7 studies, heterogeneity: *I*^*2*^ = 98.10%, *P* = 0.000, WMD = 931.50, 95% CI = 586.94 to 1276.06, *P* < 0.001, Fig. 6D).

### Left ventricular ejection fraction (LVEF)

**Fig. 7:**
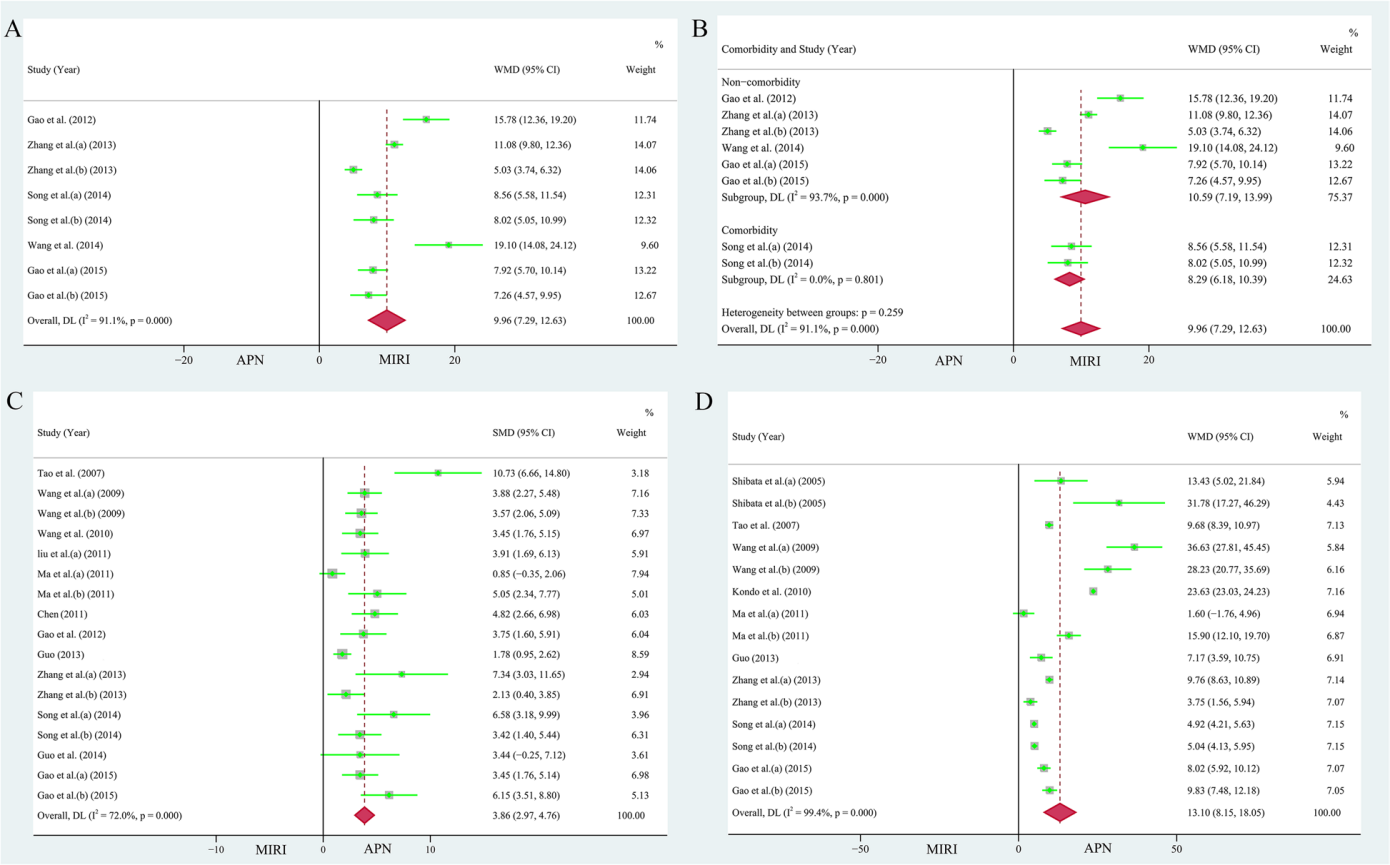
**A** Forest plot of the protective effect of APN on LVEF. **B** Subgroup analysis of the protective effect of APN on LVEF (stratified by presence of comorbidities). **C** Forest plot of the effect of APN on caspase-3 expression. **D** Forest plot of the effect of APN on apoptosis

A pooled analysis of five papers (eight study cohorts) showed that APN improved LVEF [35, 37, 39–41], with an increase of 9.96% compared to the MIRI group (n = 136, heterogeneity: *I*^*2*^ = 91.10%,* P* = 0.000, WMD = 9.96, 95% CI = 7.29 to 12.63, *P* < 0.001, Fig. 7A). Egger's test (*P* = 0.290 > 0.05, *t* = 1.17) and the funnel plot (Supplementary Fig. 3B) showed no bias. Begg's test (*P* = 0.266, *Z* = 1.11) also indicated no bias. Sensitivity analysis indicated the results were robust and reliable (Supplementary Fig. 3D).

When stratifying the studies according to the presence of comorbidities, the increasing effect of APN on LVEF persisted (non-comorbidity: 112 animals in 6 studies, heterogeneity: *I*^*2*^ = 93.70%, *P* = 0.000, WMD = 10.59, 95% CI = 7.19 to 13.99, *P* < 0.001; comorbidity: 24 animals in 2 studies, WMD = 8.29, 95% CI = 6.18 to 10.39, Fig. 7B, *P*< 0.001). However, heterogeneity was insignificant in the comorbidity subgroup (*I*^*2*^ = 00.00%, *P* = 0.801). Because the APN administration modalities and species included in the study were the same, the results were not stratified.

### Cardioprotective mechanisms of APN

#### Anti-apoptosis

The combined analysis of 12 published studies (consisting of 17 study cohorts) [27, 28, 30–33, 35–39, 41] revealed that the APN group had a lower expression level of caspase-3 (n = 252, *P* < 0.001, heterogeneity: *I*^*2*^ = 72.00%, *P* = 0.000, SMD = 3.86, 95% CI = 2.97 to 4.76, Fig. 7C). Egger's test (*t* = 6.07, *P* = 0.000 < 0.05) and the funnel plot (Supplementary Fig. 4A) showed publication bias in the selected studies. Sensitivity analysis indicated the results were robust and reliable (Supplementary Fig. 4C).

Analysis of nine papers (15 study cohorts) [26–29, 33, 36, 37, 39, 41] showed that the application of APN resulted in a significant reduction in the number of apoptotic cells (n = 190, heterogeneity: *I*^*2*^ = 99.40%, *P* = 0.000, WMD = 13.10, 95% CI = 8.15 to 18.05, *P* < 0.001, Fig. 7D). Egger's test (*P* = 0.597 > 0.05, *t* = -0.54) and the funnel plot (Supplementary Fig. 4B) indicated that there was no bias in publishing. Sensitivity analysis indicated that the results were reliable (Supplementary Fig. 4D).

### Anti-oxidation

**Fig. 8:**
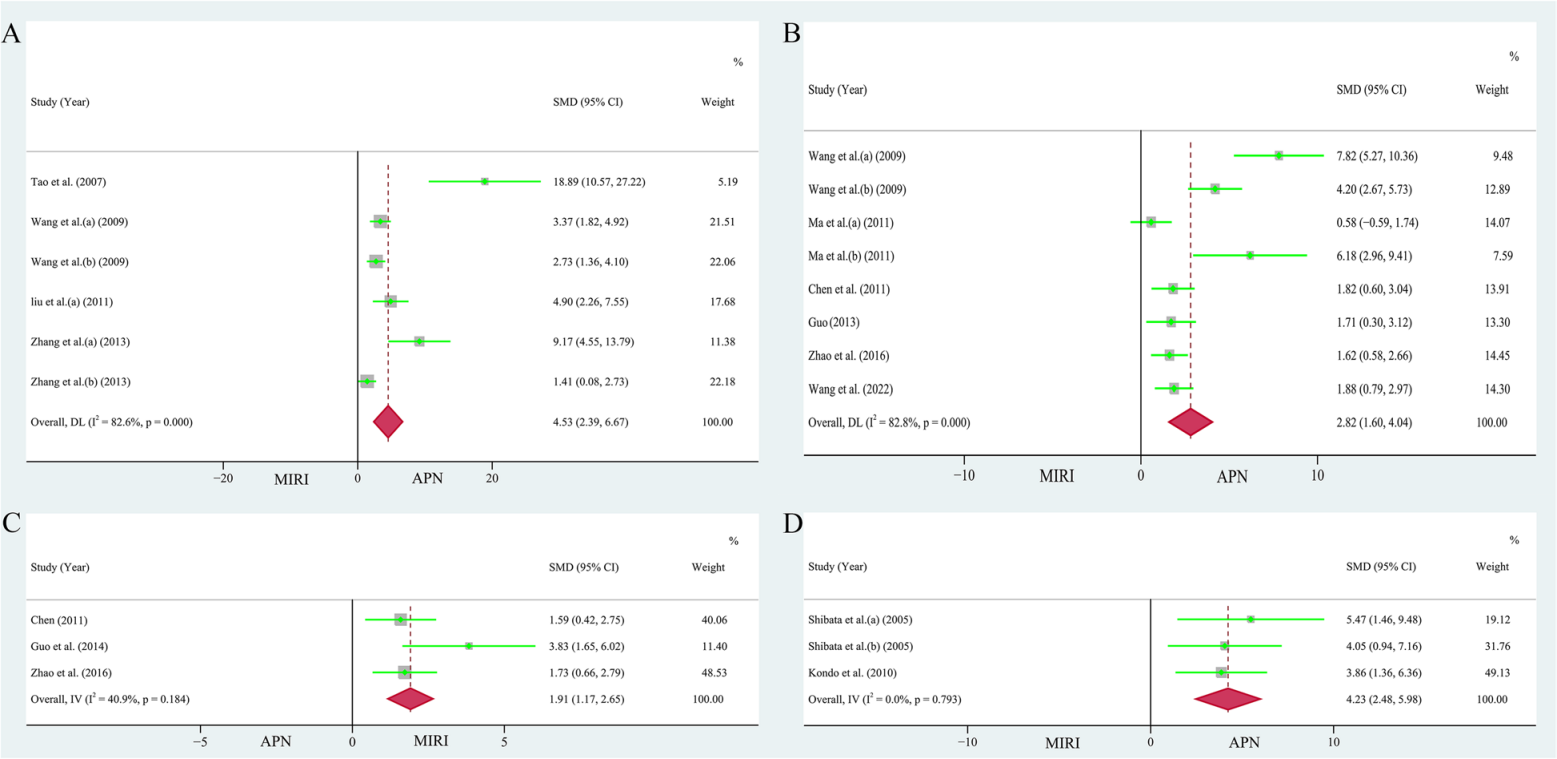
**A** Forest plot of APN effects on superoxide production. **B** Forest plot of the effect of APN on LDH content. **C** Forest plot of the effect of APN on SOD content. **D** Forest plot of the effect of APN on TNF-α levels

A pooled analysis of four papers (6 study cohorts) [27, 28, 32, 37] showed that the use of APN significantly reduced superoxide production in the myocardium, and the results were meaningful (n = 86, heterogeneity: *I*^*2*^ = 82.60%, *P* = 0.000, SMD = 4.53, 95% CI = 2.39 to 6.67, *P* < 0.001, Fig. 8A). Pooled examination of the three studies [31, 38, 42] showed that superoxide dismutase content was significantly higher in the APN cohort than in the MIRI cohort (n = 48, heterogeneity: *I*^*2*^ = 40.90%, *P* = 0.184, SMD = 1.91, 95% CI = 1.17 to 2.65, *P* < 0.001, Fig. 8C).

For studies evaluating the effect of APN on superoxide levels in the myocardium, Egger's test (*P* = 0.003 < 0.05, *t* = 6.39) and the funnel plot (Supplementary Fig. 5A) showed publication bias in the selected studies. However, sensitivity analysis suggested that the results were reliable (Supplementary Fig. 5C).

### Anti-inflammatory

The combined examination of six articles (consisting of 8 study cohorts) [28, 33, 34, 36, 42, 45] revealed a significant decrease in lactate dehydrogenase (LDH) levels within the APN group (n = 140, heterogeneity: *I*^*2*^ = 82.80%, *P* = 0.000, SMD = 2.82, 95% CI = 1.60 to 4.04, Fig. 8B, *P* < 0.001). Analysis of two papers (3 study cohorts) [26, 29] showed that APN application significantly reduced TNF-α levels (n = 26, heterogeneity: *I*^*2*^ = 00.00%, *P* = 0.793, SMD = 4.23, 95% CI = 2.48 to 5.98, *P* < 0.001, Fig. 8D).

For the studies on the effect of APN on lactate dehydrogenase levels, Egger's test (*P* = 0.009 < 0.05, *t* = 3.81) and the funnel plot (Supplementary Fig. 5B) showed publication bias in the selected studies. However, the reliability of the results was supported by the sensitivity analysis (Supplementary Fig. 5D).

## Discussion

The present meta-analysis included 20 studies, containing 426 animals, and the studies were high quality. The risk of bias was low, and the results were reliable. Pooled analyses showed that APN significantly reduces the size of the infarcted myocardium, regulates cardiovascular function, and suppresses the concentration of biomarkers associated with myocardial infarction. APN also improves myocardial inflammation, oxidative stress, and apoptosis.

MIRI is unavoidable in cardiac surgery, and the incidence increases yearly. Rescue from this injury remains a substantial challenge due to the lack of effective treatments [46]. The protective effect of APN on MIRI has not been reported by meta-analyses, and most of the evidence has been derived from basic science research. Interestingly, the results of some studies suggest different mechanisms. Some researchers have suggested that APN benefits MIRI through its adenine monophosphate-activated protein kinase signaling pathways [43, 47, 48]. However, some scholars believe that the cardioprotective effect of APN is not dependent on this mechanism [28, 49]. The protective effect of APN on MIRI may involve several mechanisms. A large body of evidence suggests that APN exerts cardioprotective functions through multiple molecular mechanisms [50, 51]. Understanding these protective mechanisms will help to better understand adiponectin. The currently reported mechanisms of action of APN in MIRI involve the mechanisms described below.

- (1) Modulation of angiogenesis: Enhancing blood vessel formation and restoring blood supply is essential for clearing inflammation and repairing myocardial damage. APN, a chemotactic factor, has the ability to stimulate the transformation of endothelial cells into structures resembling capillaries in a laboratory setting, thereby controlling the process of angiogenesis [52]. APN ameliorates vascular damage after ischemic stress through an AMP-activated protein kinase-dependent pathway (AMPK) [53].
- (2) Regulating autophagy: In recent years, autophagy has been recognized as one of the critical mechanisms underlying the cardioprotective effects of APN. The ERK/mTOR/AMPK signaling pathway regulates autophagy in cells, thereby protecting cardiomyocytes from oxidative stress [54]. In addition, APN also triggers autophagy in macrophages through the AMPK pathway and suppresses the inflammatory reaction, resulting in a reduction in cardiac fibrosis [55].
- (3) Controlling the metabolism of fats and sugars: Numerous studies have indicated a connection among inflammation, oxidative stress, and disorders in the processing of glucose and lipids, ultimately affecting cardiovascular balance and damaging heart muscle [10, 56]. Protection of pancreatic β cells by APN enhances the absorption and utilization of monosaccharides by tissues, reduces sugar production, and regulates glucose metabolism [50]. APN also promotes adipocyte differentiation and facilitates fatty acid oxidation and turnover, thereby protecting the myocardium from injury [57].

Several studies have reported that APN is associated with cardiovascular disease development. Tentolouris et al. found that plasma lipoprotein levels are inversely proportional to the risk of developing IDH [58]. Numerous epidemiologic studies have demonstrated that plasma APN levels are significantly lower after myocardial infarction in obese and type 2 diabetic patients [59, 60]. It has been demonstrated that the degree of intimal hyperplasia after arterial injury is twice as high in APN knockout mice as in wild-type mice, whereas this process is markedly inhibited by supplementation with exogenous APN [61]. These findings suggest that APN is an anti-MIRI agent. Several sensitivity analyses and subgroup analyses have supported these results. In the present meta-analysis, sensitivity testing found that no studies had an impact on the results of the analysis, suggesting that the present results were stable, significant, and of high quality. Exogenous APN showed better therapeutic effects than other forms of APN. Fruebis et al. reported that exogenous APN releases more fatty acids and has a faster and more effective treatment effect in vivo after a single injection compared to full-length APN [62]. In addition, exogenous APN supplementation may be superior to full-length APN in renal insufficiency because exogenous APN lacks the NH_2_-terminal structural domain and does not bind to and inactivate cystatin C [63, 64].

Because APN has a better safety profile and lower toxicity than other monomers, it is necessary to continue to explore and support clinical trials. Moreover, the targets of APN for the treatment of MIRI should be explored in future studies to improve its molecular mechanism to accelerate the clinical process.

### Strengths and limitations

The present meta-analysis is the first to elucidate the therapeutic effect of APN in MIRI based on animal experiments and establish a foundation for MIRI treatment with APN. The sample size of this meta-analysis was large, and many bias and sensitivity analyses were performed, demonstrating highly credible conclusions. In addition, the relevant mechanisms were summarized and elaborated, and the protective role of APN in MIRI was demonstrated, providing a basis for future research.

Although the number of animals in the present study was large and the safety and validity of the results were adequate, there were some limitations. First, the amount of data obtained from the animals was relatively small and could not be analyzed in depth. Second, due to the paucity of relevant studies, we were unable to determine whether the cardioprotective effects of APN are enhanced with further dose increases based on dose–response modeling in animal studies. Third, the results of the pooled analyses using SMD values with 95% CI should be interpreted with caution. SMD is a relative indicator and may not reflect the actual event. Fourth, only animal studies were included because only a few pertinent clinical studies have been published. In addition, the development of MIRI in real-world clinical settings is complex, thus limiting the present findings. Finally, meaningful results are easier to publish, indicating a likely overestimation of the efficacy of APN.

## Conclusion

The present meta-analysis was based on preclinical studies and systematically illustrated the value of APN anti-MIRI. APN protects damaged myocardium by reducing the size of myocardial infarction and improving intracardiac hemodynamics. Moreover, APN has multiple mechanisms of action, including its ability to reduce inflammation, prevent cell death, and counteract oxidative stress. This conclusion holds despite limitations that reduce the persuasiveness of the evidence. APN is a promising anti-MIRI substance that may be incorporated into the treatment of MIRI and provide a strategy for MIRI treatment.

### Supplementary Information

**Additional file 1:** **Supplementary File 1.** PRISMA 2020 Checklist. **Supplementary Figure 1.** Funnel plot and sensitivity analysis of myocardial infarction size. **Supplementary Figure 2.** Funnel plot and sensitivity analysis. (A and C) LVEDP, (B and D) +dp/dtmax. **Supplementary Figure 3.** Funnel plot and sensitivity analysis. (A and C) -dp/dtmax, (B and D) LVEF. **Supplementary Figure 4.** Funnel plot and sensitivity analysis. (A and C) Caspase-3, (B and D) TUNEL-positive cells. **Supplementary Figure 5.** Funnel plot and sensitivity analysis. (A and C) Superoxide content, (B and D) LDH.

## Data Availability

The original contributions shown in the study are selected in the article/supplementary material. Appropriate inquiries can be contacted with the corresponding author.

## Abbreviations

IHD: Ischemic heart disease
CVD: Cardiovascular disease
PCI: Percutaneous coronary intervention
CABG: Coronary artery bypass grafting
MIRI: Myocardial ischemia–reperfusion injury
APN: Adiponectin
I2: I squared
CI: Confdence interval
SMD: Standardized mean difference
WMD: Weighted mean difference
IV: Intravenous
IP: Intraperitoneal
LAD: Left anterior descending
LVEDP: Left ventricular end-diastolic pressure
+ dp/dtmax: Maximum rate of left ventricular pressure rise
-dp/dtmax: Maximum rate of left ventricular pressure decrease
LVEF: Left ventricular ejection fraction
SOD: Superoxide dismutase
PROSPERO: Pravastatin in elderly individuals at risk of vascular disease
PRISMA: Preferred Reporting Items for Systematic Reviews and Meta-Analyses
SYRCLE: Systematic Review Center for Laboratory animal Experimentation
GRADE: Grading of Recommendations, Assessment, Development, and Evaluations
