# Supplementary material for: Adiponectin protects against myocardial ischemia–reperfusion injury: a systematic review and meta-analysis of preclinical animal studies
Source: Lipids Health Dis. 2024 Feb 17;23:51. doi: 10.1186/s12944-024-02028-w (PMC10874037; doi:10.1186/s12944-024-02028-w)
Supplement: Supplementary file 1 — Additional file 1: Supplementary File 1. PRISMA 2020 Checklist. Supplementary Figure 1. Funnel plot and sensitivity analysis of myocardial infarction size. Supplementary Figure 2. Funnel plot and sensitivity analysis. (A and C) LVEDP, (B and D) +dp/dtmax. Supplementary Figure 3. Funnel plot and sensitivity analysis. (A and C) -dp/dtmax, (B and D) LVEF. Supplementary Figure 4. Funnel plot and sensitivity analysis. (A and C) Caspase-3, (B and D) TUNEL-positive cells. Supplementary Figure 5. Funnel plot and sensitivity analysis. (A and C) Superoxide content, (B and D) LDH. [file 12944_2024_2028_MOESM1_ESM.docx]

**Supplementary material**

Supplementary File 1 | PRISMA 2020 Checklist


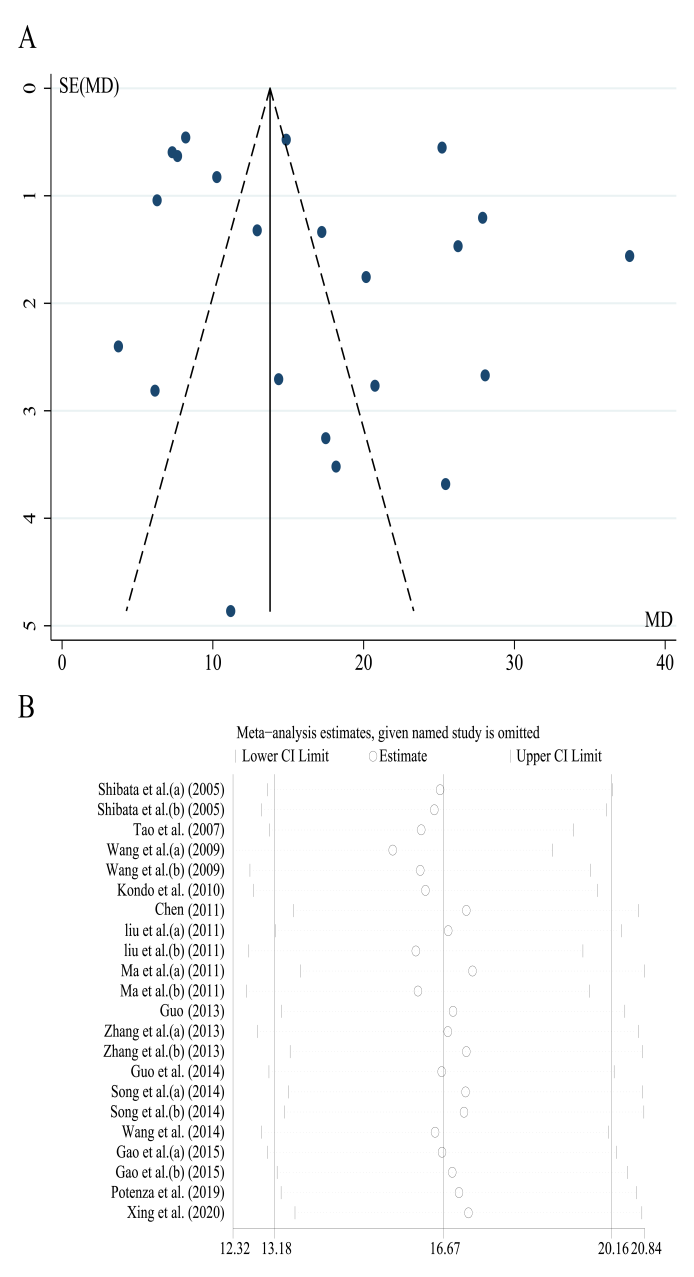


Supplementary Figure 1 | Funnel plot and sensitivity analysis of myocardial infarction size.

Supplementary Figure 2 | Funnel plot and sensitivity analysis. (A and C) LVEDP, (B and D) +dp/dtmax.

Supplementary Figure 3 | Funnel plot and sensitivity analysis. (A and C) -dp/dtmax, (B and D) LVEF.


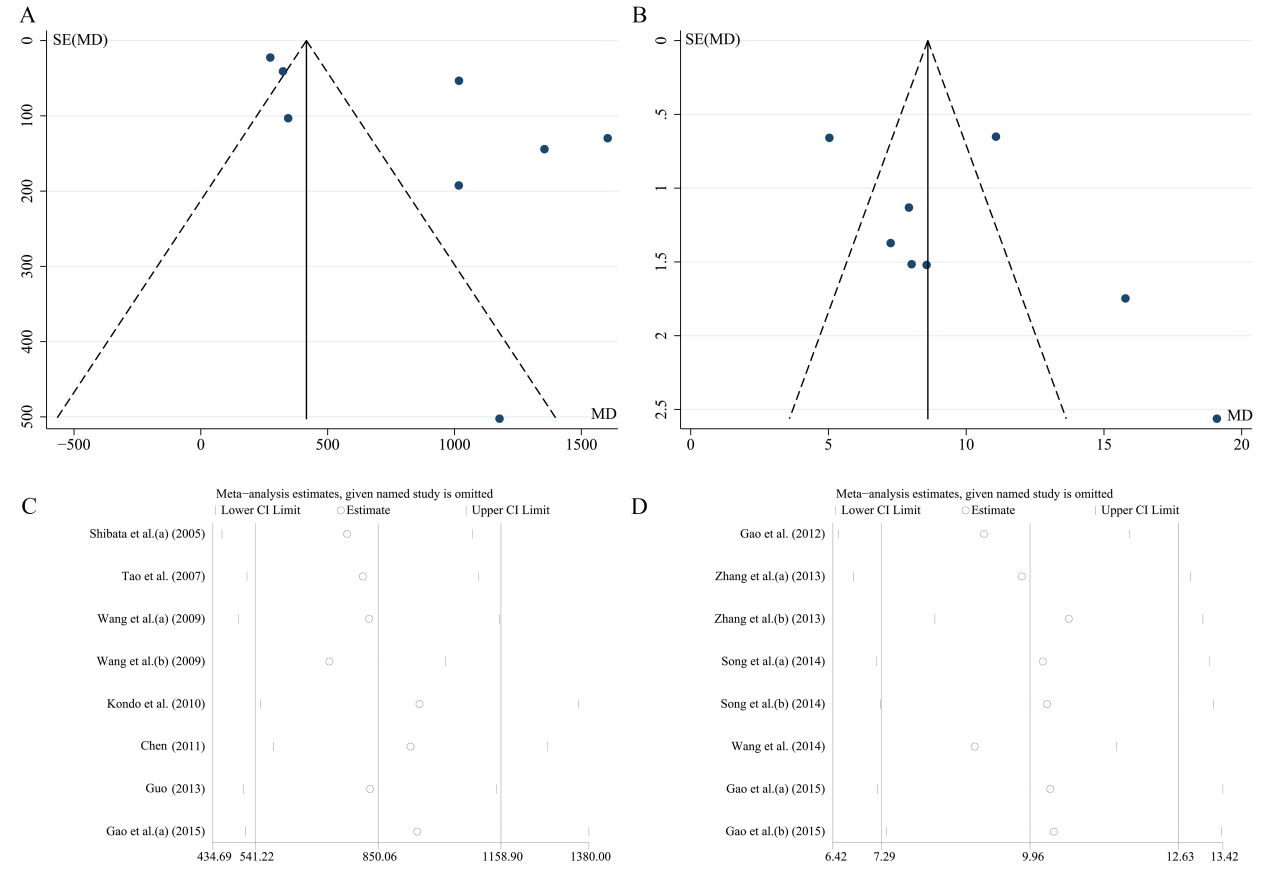


Supplementary Figure 4 | Funnel plot and sensitivity analysis. (A and C) Caspase-3, (B and D) TUNEL-positive cells.


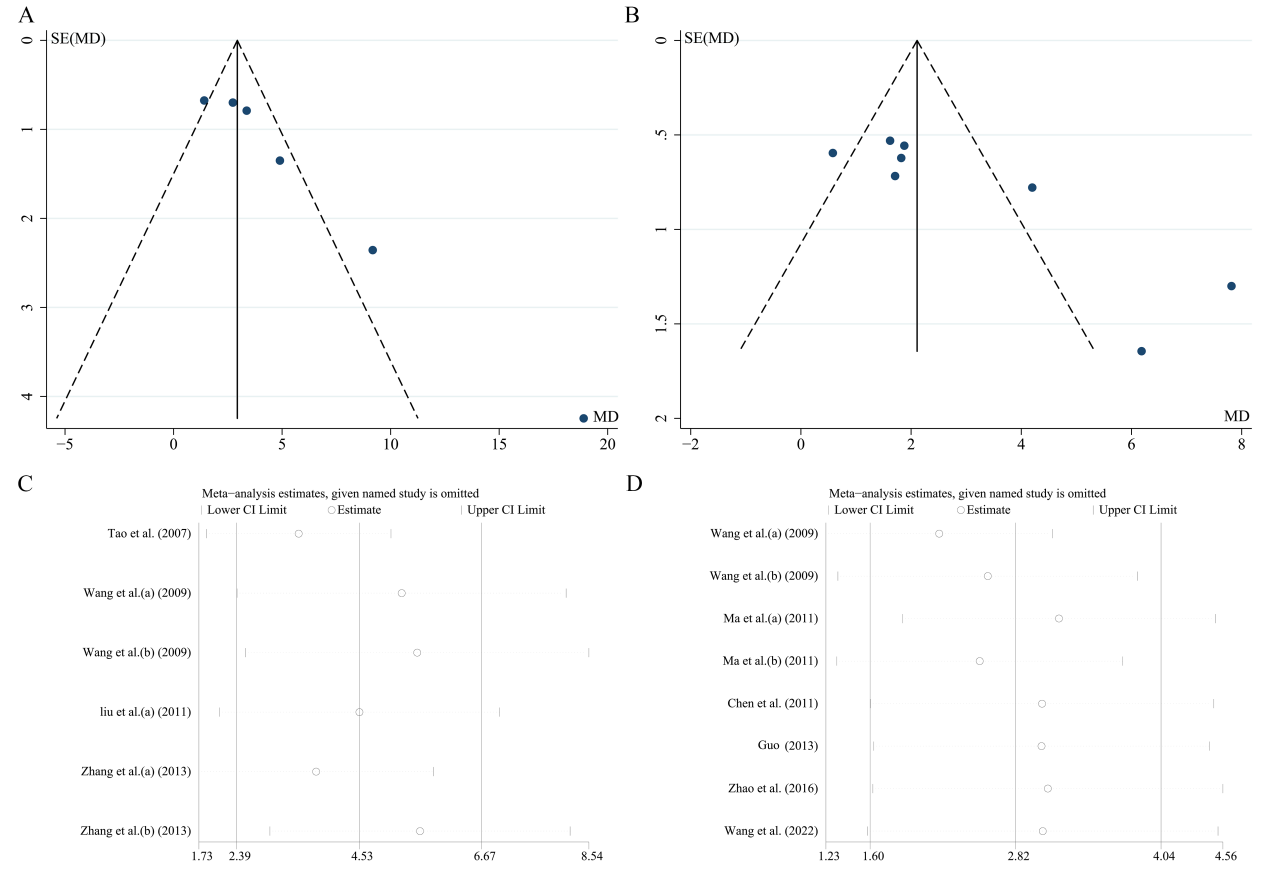


Supplementary Figure 5 | Funnel plot and sensitivity analysis. (A and C) Superoxide content, (B and D) LDH.
